# Eliminating endotoxin by polymyxin B hemoperfusion and/or continuous renal replacement therapy: should the focus be on timing, dosing, and type of renal epuration?

**DOI:** 10.1186/s13613-019-0512-0

**Published:** 2019-03-08

**Authors:** Patrick M. Honore, David De Bels, Sebastien Redant, Herbert D. Spapen

**Affiliations:** 1 ICU Department, Centre Hospitalier Universitaire Brugmann-Brugmann University Hospital, Place Van Gehuchtenplein, 4, 1020 Brussels, Belgium; 2 Ageing and Pathology Research Group, Vrije Universiteit Brussel, Brussels, Belgium

In a recent issue of Annals of Intensive Care, Navas et al. reported their experience with adjuvant polymyxin B hemoperfusion (PMX-HP) therapy in suspected Gram-negative septic shock [1]. PMX-HP is an extracorporeal technique that selectively adsorbs and eliminates endotoxin from the circulation [2]. Although theoretically only beneficial in endotoxin-driven Gram-negative sepsis, effective PMX-HP treatment does not depend upon type of microorganism or infection site. PMX-HP may offer a particular survival benefit in patients with plasma endotoxin activity (EA) levels between 0.6 and 0.9 EU/mL [3].

The study of Navas et al. is original because it compared for the first time the use of continuous renal replacement therapy (CRRT) plus PMX-HP with CRRT alone in patients who potentially would benefit most from such treatment (i.e., carefully selected type of infection, presence of multi-organ failure, and an EA cutoff level > 0.6 EU/mL as predetermined “biomarker”). All CRRT/PMX-HP patients underwent hemofiltration through an acrylonitrile (AN) 69 membrane. The main study conclusion was that adding PMX-HP to CRRT resulted in faster decrease of EA without improving respiratory, hemodynamic, biological, and outcome parameters [1].

The authors deserve all respect for their tenacity in conducting the study (it took nearly 3.5 years to recruit 18 patients!) but also for mustering an impressive amount of clinical and laboratory data. Obvious study weaknesses are the paucity of patients, an excess mortality at day 2 in the CRRT-only group, the failure to recruit pure Gram-negative infection in half of the population, and the lack of information regarding individual fluid balances. Also, differences in antibiotic-induced endotoxin-liberating potential might have caused less endotoxin load in the almost exclusively meropenem-treated CRRT-only patients [4].

Binding of circulating endotoxin by the PMX-HP column may decrease endotoxin levels by up to 90% after two treatments [2]. Therefore, it is remarkable that the EA level after completion of PMX-HP therapy was still high and not different from that obtained with CRRT treatment alone (0.59 vs. 0.57 EU/mL). Fast removal of endotoxin as the prime culprit associated with severity and mortality of sepsis is a reasonable therapeutic approach [5], but it remains to be proven whether it will interrupt or modulate an already ongoing inflammatory cascade. Despite comparable disease severity, degree of organ failure, and baseline EA levels in both treatment arms of the Navas study, the cytokine profile suggested that inflammation was more pronounced in patients only undergoing CRRT. Moreover, pretreatment levels of interleukin-6, a “distal” cytokine that intensifies and perpetuates the inflammatory response, were significantly lower in CRRT/PMX-HP patients as compared with CRRT-treated subjects. This implies that CRRT/PMX-HP was probably started at an earlier stage of septic shock rendering the hemoperfusion component more effective. Differences in inflammatory state and time of introducing hemoperfusion may have accounted for the observed high mortality within the first days of treatment in the CRRT group.

The standard use of PMX-HP applied as a 2-h session on 2 consecutive days may be challenged. Endotoxin release indeed is a dynamic process characterized by a continuous production at the site of infection but also by leakage from the gut reservoir. Mitaka et al. showed that a 24-h PMX-HP treatment removed endotoxin at a rate of 74.4% in septic shock patients [6] suggesting a role for “prolonged” PMX-HP approach. Navas et al. applied hemofiltration with an AN69 membrane which has no endotoxin-adsorptive capacity. The modified oXiris AN69 membrane has a surface polarity that facilitates adsorption of endotoxin. This membrane exhibits in vitro removal capacities for endotoxin that match those of PMX-HP [7]. In addition, oXiris AN69 effectively adsorbs a wide range of inflammatory cytokines and mediators [8]. The theoretical usefulness of applying CRRT using the oXiris AN69 membrane has not yet been assessed clinically except for a small study in Gram-negative sepsis-induced acute kidney injury, reporting reduced organ failure in patients undergoing continuous veno-venous hemofiltration with this particular hemofilter [9].

The data reported by Navas et al. contribute to creating a backbone for further investigation of PMX-HP/CRRT treatment in appropriately selected patients. However, future studies need to carefully consider aspects of timing and duration of therapy. Blood purification techniques that combine endotoxin removal with elimination of relevant inflammatory mediators may be a promising option.

## Abbreviations

PMX-HP: polymyxin B hemoperfusion
EA: endotoxin activity
CRRT: continuous renal replacement therapy
AN: acrylonitrile
